# Supplementary material for: C3d-Positive Preformed DSAs Tend to Persist and Result in a Higher Risk of AMR after Kidney Transplants
Source: J Clin Med. 2020 Jan 30;9(2):375. doi: 10.3390/jcm9020375 (PMC7073748; doi:10.3390/jcm9020375)
Supplement: Supplementary file 1 [file jcm-09-00375-s001.pdf]

Table S1. Donor-specific antibody characteristics in groups 2 and 4 recipients.

Characteristics	Group 2: Cryptic pDSA rebound		*p*-value	Group 4: pDSA persistent		*p*-value
	**Post-Pdsa C3d (-)**	Post-pDSA C3d (+)		Post-pDSA C3d (-)	Post-pDSA C3d (+)	
**Number**	**15**	4		28	9	
R age, years (IQR)	49.0 (40.0 – 56.3)	45.5 (39.8 – 61.1)	0.920	53.0 (47.4 – 59.6)	51.0 (48.0 – 61.0)	0.859
R Sex (male) (%)	5 (33.3)	2 (50.0)	0.603	9 (32.1)	3 (33.3)	1.000
Dialysis duration, day (IQR)	1074.0 (52.2 – 2340.2)	75.0 (24.6 – 318.5)	0.161	1696.0 (202.7 – 2655.7)	95.0 (25.3 – 807.0)	0.035
Underlying disease (%)						
DM	2 (13.3)	1 (25.0)	0.126	4 (14.3)	1 (11.1)	1.000
GN (1~3)	0 (0.0)	1 (25.0)		6 (21.4)	2 (22.2)	
IgA	3 (20.0)	1 (25.0)		2 (7.1)	1 (11.1)	
Other	10 (66.7)	1 (25.0)		16 (57.1)	5 (55.6)	
Re-transplantation (%)	3 (20.0)	1 (25.0)	1.000	11 (39.3)	3 (33.3)	1.000
DDKT (%)	6 (40.0)	3 (75.0)	0.303	13 (46.4)	5 (55.6)	0.714
D age, years (IQR)	41.0 (31.8 – 51.8)	44.5 (35.9 – 56.6)	0.841	45.5 (33.0 – 51.0)	53.0 (30.7 – 56.0)	0.348
D Sex (male) (%)	10 (66.7)	2 (50.0)	0.603	21 (75.0)	7 (77.8)	1.000
Desensitization						
RTX	0 (0.0)	0 (0.0)		25 (89.3)	8 (88.9)	
RTX + PP	0 (0.0)	0 (0.0)		3 (10.7)	1 (11.1)	
Induction therapy						
ATG	7 (46.7)	3 (75.0)		27 (96.4)	9 (100.0)	
Basiliximab	8 (53.3)	1 (25.0)		1 (3.6)	0 (0.0)	
Maintenance regimen						
CsA + MMF (PD)	0 (0.0)	0 (0.0)		0 (0.0)	0 (0.0)	
FK + MMF (PD)	14 (93.3)	4 (100.0)		28 (100.0)	9 (100.0)	
Sirolimus/Everolimus combination	1 (6.7)	0 (0.0)		0 (0.0)	0 (0.0)	
Pre-sensitization (PRA %)						
Class I	0.0 (0.0 – 0.0)	17.0 (5.8 – 34.0)	0.028	69.0 (2.5 – 90.2)	51.0 (0.0 – 66.3)	0.283
Class II	0.0 (0.0 – 11.7)	7.0 (0.0 – 16.9)	0.585	30.5 (0.0 – 74.6)	36.0 (27.3 – 68.3)	0.365
HLA mismatches, (IQR)	3.0 (2.0 – 4.0)	5.0 (2.1 – 5.6)	0.125	3.0 (2.0 – 4.0)	4.0 (2.0 – 4.3)	0.116
Pre-transplant pDSA (%)						
Class I only	NA	NA		13 (68.4)	0 (0.0)	
Class II only	NA	NA		9 (46.4)	5 (55.6)	
Class I+II	NA	NA		6 (21.4)	4 (44.4)	
MFI of pre-pDSA*						
Class I	NA	NA	NA	1993.0 (1478.0 - 3078.2)	2486.0 (1259.8 - 891.7)	0.935
Class II	NA	NA	NA	4239.0 (2850.8 - 5025.0)	7585.0 (4216.3 - 3875.0)	0.053
C3d binding activity of pre-DSA						

Class I	NA	NA	NA	0 (0.0)	0 (0.0)	
Class II	NA	NA		3 (10.7)	5 (55.6)	
Post-transplant pDSA (%)						
Class I only	6 (40.0)	1 (25.0)		11 (39.3)	0 (0.0)	
Class II only	7 (46.7)	3 (75.0)		14 (50.0)	7 (77.8)	
Class I+II	2 (13.3)	0 (0.0)		3 (10.7)	2 (22.2)	
MFI of post-pDSA*						
Class I	1958.0 (839.3 – 6077.8)	10273.0 (10273.0 – 10273.0)	0.121	1071.0 (831.0 – 1693.0)	3722.5 (1626.0 – 5819.0)	0.164
Class II	1344.0 (684.3 – 13589.0)	5899.0 (4650.7 – 12355.7)	0.309	1520.0 (1007.0 – 3144.3)	11397.0 (7559.3 – 14884.7)	0.001

All continuous variables are presented as the median and IQR. * Median of maximum DSA MFI.***p*-values among the four groups without consideration of the post-DSA C3d-binding capacity. R, recipient; IQR, interquartile range; DM, diabetes mellitus; GN, glomerulonephritis; IgA, IgA nephropathy; DDKT, deceased donor kidney transplantation; D, donor; pDSA, preformed donor-specific HLA antibody; NA, not applicable; MFI, mean fluorescence intensity; RTX, rituximab; PP, plasmapheresis; rATG, recombinant anti-thymocyte globulin; CsA, cyclosporine A; MMF, mycophenolate mofetil; PD, prednisolone; FK, FK506; PRA, panel reactive antibodies.

Table S2. MFI of 105 pDSAs from 75 recipients.

Group	Number	Median MFI of pre-pDSAs (IQR)				Median MFI of post-pDSAs (IQR)			
**Group 2: Cryptic pDSA rebound**	**24**	**NA**				1665.5	(818.4	–	9378.8)
Post-C3d (-)	20	NA				1276.0	(762.8	–	7941.6)
Post-C3d (+)	4	NA				8086.0	(5025.2	–	12241.2)
**Group 3: pDSA reversed**	37	1500.0	(1161.3	–	2533.0)	NA			
**Group 4: pDSA persistent**	44	3524.5	(1599.8	–	5375.9)	1629.0	(969.3	–	5634.8)
Post-C3d (-)	34	2584.5	(1496.0	–	4446.3)	1371.0	(833.3	–	1901.8)
Post-C3d (-), pre-C3d (-)	32	2323.5	(1484.0	–	4139.4)	1277.5	(829.3	–	1767.9)
Post-C3d (-), pre-C3d (+)	2	9905.5	(9659.0	–	10152.0)	6814.5	(3106.0	–	10523.0)
Post-C3d (+)	10	8846.5	(4492.8	–	13452.0)	9874.0	(7284.8	–	14296.9)
Post-C3d (+), pre-C3d (-)	4	4032.0	(2031.2	–	5091.3)	7509.0	(6202.4	–	10644.9)
Post-C3d (+), pre-C3d (+)	6	11725.0	(9897.8	–	15149.5)	12749.0	(8195.1	–	17251.3)

IQR, interquartile range; pDSA, preformed donor-specific HLA antibody; NA, not applicable; MFI, mean fluorescence intensity.

Table S3. Distribution of mean fluorescence intensity of 1,522 anti-HLA antibodies in a single antigen bead assay according to their C3d-binding capacity and optimal cut-offs to predict C3d-binding capacity.

**Class**	**Locus**	**Total**		C3d (+)		C3d (-)		*p*-value	Optimal cut-off (MFI)
		**Number**	Median MFI (IQR)	Number (%)	Median MFI (IQR)	Number (%)	Median MFI (IQR)		
**Class I**	**Total**	**919**	2244.0 (1370.5 – 4041.7)	139 (15.1)	9429.0 (5457.2 – 16016.2)	780 (84.9)	1988.0 (1270.7 – 3190.2)	< 0.001	7797
	A	322	2753.5 (1453.3 – 5223.3)	80 (24.8)	8213.0 (4998.0 – 16243.2)	242 (75.2)	2230.5 (1257.8 – 3582.0)	< 0.001	6236
	B	526	2136.5 (1339.6 – 3573.4)	59 (11.2)	11002.0 (7080.5 – 14012.2)	467 (88.8)	1966.5 (1268.2 – 3027.5)	< 0.001	9026
	C	71	1798.0 (1339.0 – 2916.8)	0 (0)	NA	71 (100)	1798.0 (1339.0 – 2916.8)	NA	NA
Class II	Total	596	4021.0 (1450.9 – 10361.3)	254 (42.6)	10341.0 (6692.7 – 14206.8)	342 (57.4)	1711.0 (1001.6 – 3400.8)	< 0.001	4460
	DR	370	4585.0 (1485.3 – 10032.7)	171 (46.2)	9601.5 (6042.0 – 12839.4)	199 (53.8)	1702.0 (896.2 – 3709.5)	< 0.001	3848
	DQB1	159	6860.0 (2460.0 – 14600.3)	73 (45.9)	14101.0 (10030.0 – 16986.3)	86 (54.1)	2593.5 (1510.1 – 4399.1)	< 0.001	4460
	DPB1	67	1127.0 (880.2 – 2564.2)	10 (14.9)	6705.0 (2965.7 – 10358.7)	57 (85.1)	1082.0 (816.3 – 1549.3)	< 0.001	4027

MFI, mean fluorescence intensity; IQR, interquartile range; NA, not applicable.

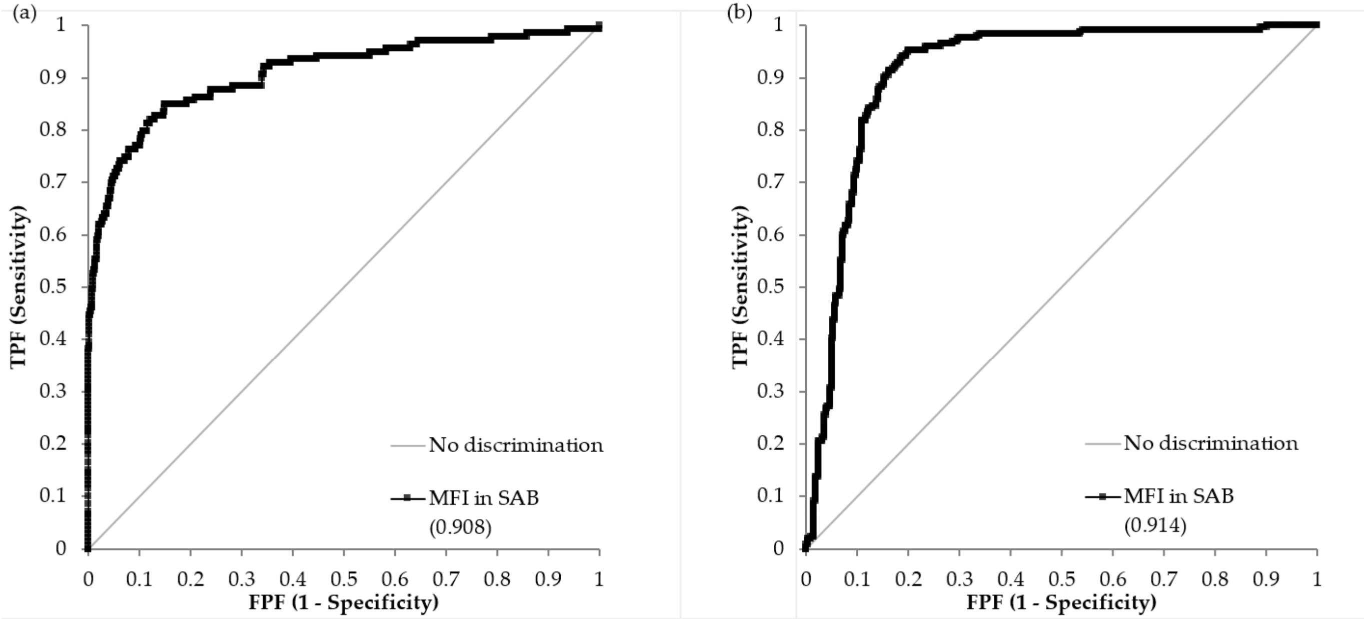

	AUC	95% CI	*p*-value
MFI in SAB	0.908	0.876 - 0.941	< 0.001

	AUC	95% CI	*p*-value
MFI in SAB	0.914	0.889 - 0.938	< 0.001

Figure S1. Receiver operating curves analysis of the mean fluorescence intensity of single antigen bead-based antibody identification assay performance in an effort to predict complement binding capability in class I (a) and class II (b); TPF, true-positive fraction; FPF, false-positive fraction; AUC, area under the curve; CI, confidence interval; MFI, mean fluorescence intensity; SAB, single antigen bead-based identification assay.

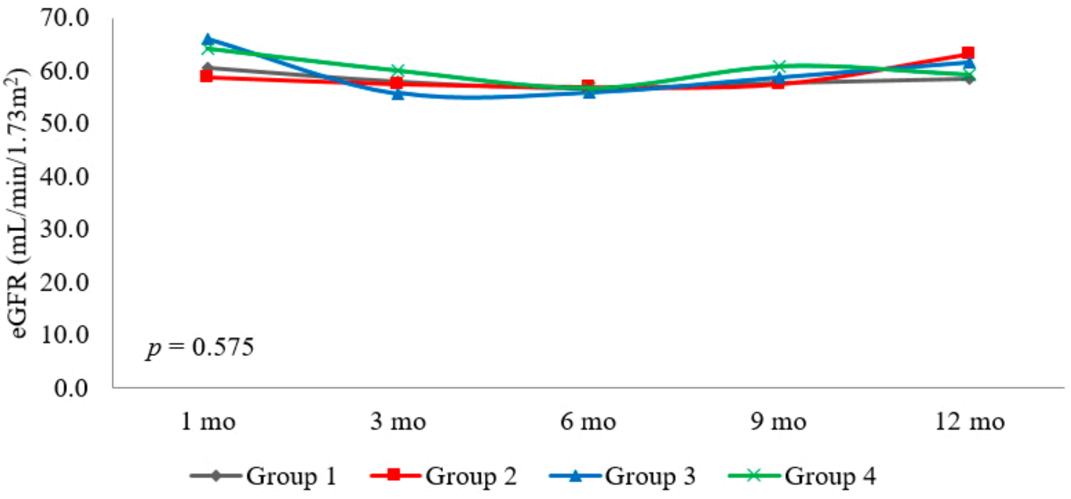

Figure S2. eGFR differences observed among the groups were not significantly different. eGFR was calculated by the modification of diet in renal disease (MDRD) study equation.; eGFR, estimated glomerular filtration rate.
